# Reduced Liver Lipid Peroxidation in Subcellular Fractions Is Associated with a Hypometabolic State in Rats with Portacaval Anastomosis

**DOI:** 10.1155/2019/4565238

**Published:** 2019-02-21

**Authors:** Olivia Vázquez-Martínez, Héctor Valente-Godínez, Andrés Quintanar-Stephano, Deisy Gasca-Martínez, Mayra L. López-Cervantes, Lourdes Palma-Tirado, María de Jesús Guerrero-Carrillo, Mariela Pérez-Solís, Mauricio Díaz-Muñoz

**Affiliations:** ^1^Cellular and Molecular Department, Neurobiology Institute, Campus UNAM-Juriquilla, Querétaro 76230, Mexico; ^2^Physiology and Pharmacology Department, Basic Science Center, Autonomous University of Aguascalientes, Aguascalientes 20131, Mexico; ^3^Behavioral Analysis Unit, Neurobiology Institute, Campus UNAM-Juriquilla, Querétaro 76230, Mexico; ^4^Microscopy Unit, Neurobiology Institute, Campus UNAM-Juriquilla, Querétaro 76230, Mexico

## Abstract

A surgical connection between portal and inferior cava veins was performed to generate an experimental model of high circulating ammonium and hepatic hypofunctioning. After 13 weeks of portacaval anastomosis (PCA), hyperammonemia and shrinkage in the liver were observed. Low glycemic levels accompanied by elevated levels of serum alanine aminotransferase were recorded. However, the activity of serum aspartate aminotransferase was reduced, without change in circulating urea. Histological and ultrastructural observations revealed ongoing vascularization and alterations in the hepatocyte nucleus (reduced diameter with indentations), fewer mitochondria, and numerous ribosomes in the endoplasmic reticulum. High activity of hepatic caspase-3 suggested apoptosis. PCA promoted a marked reduction in lipid peroxidation determined by TBARs in liver homogenate but specially in the mitochondrial and microsomal fractions. The reduced lipoperoxidative activity was also detected in assays supplemented with Fe^2+^. Only discreet changes were observed in conjugated dienes. Fluorescent probes showed significant attenuation in mitochondrial membrane potential, reactive oxygen species (ROS), and calcium content. Rats with PCA also showed reduced food intake and decreased energy expenditure through indirect calorimetry by measuring oxygen consumption with an open-flow respirometric system. We conclude that experimental PCA promotes an angiogenic state in the liver to confront the altered blood flow by reducing the prooxidant reactions associated with lower metabolic rate, along with significant reduction of mitochondrial content, but without a clear hepatic dysfunction.

## 1. Introduction

Portacaval anastomosis (PCA)/Eck's fistula is a surgical manoeuvre that is widely used in clinical gastroenterology to mitigate hemodynamic alterations associated with chronic liver dysfunction such as esophageal varices [1] and hepatorenal syndrome [2]. Experimentally, PCA has been utilized as a protocol to generate hepatic encephalopathy associated with increased levels of circulating ammonium (NH_4_^+^) [3]. PCA involves closing the portal vein first by disconnecting the circulation between the duodenum and the liver, then by connecting the distal section of the portal vein to an oval window on the inferior cava vein. The consequence of this surgery is the portal blood bypassing directly to the systemic blood circulation [4]. This condition avoids the correct biochemical processing nutrients ingested by the liver and deeply alters the bioenergetic status of this organ [5].

It has been postulated that hepatic encephalopathy associated with PCA is accompanied by oxidative/nitrosative stress in cerebral components, resulting in the activation of NMDA receptors and the nitration of key enzymes in the astrocytic nitrogen-handling enzymes such as glutamine synthetase. Eventually, these alterations combined with energy disruption by manganese and ammonium participation result in neuronal circuit disruption and brain swelling [6]. In contrast, much less is known about the metabolic consequences that take place within the liver during PCA. Some reports have explored the decrease in ketogenesis [7] and the reduction in the mixed-function oxidase system [8] and lipogenic activity [9] as well as the harmful effect on the liver regenerative ability after partial hepatectomy [10].

To gain a better understanding of the effects of PCA on liver metabolic parameters, the present project was aimed at characterizing (1) the prooxidant reactions that occur in subcellular fractions by measuring the levels of conjugated dienes (CD) and thiobarbituric acid reactive substances (TBARs) as well as (2) the presence of mitochondrial ROS, the level of mitochondrial membrane potential, and mitochondrial Ca^2+^ content by using fluorescent techniques. Biochemical parameters were complemented with (3) histological and ultrastructural observations. In addition, (4) rats with PCA surgery were placed in metabolic cages to evaluate their metabolic performance by indirect calorimetric techniques (respirometry). The results showed significant metabolic and structural adaptations of the liver indicating a vascularization process and a reduction in the metabolic rate as consequences of PCA.

## 2. Materials and Methods

### 2.1. Experimental Protocol

The experiments were performed with male Wistar rats weighing approximately 280 g (~8 weeks old) at the beginning of the experiment. The animals were put in individual cages (17 × 41 × 20 cm) at room temperature (~22°C) and maintained in a 12 h light:12 h darkness cycle (light on at 08:00 h). Access to food and water was *ad libitum*. Rats were divided into 2 groups according to the surgical procedure: placebo surgery (sham) and portacaval anastomosis (PCA). All experimental procedures were approved and conducted in accordance with the institutional guide for care and use of animals under biomedical experimentation and under international ethical standards (Universidad Nacional Autónoma de México).

### 2.2. Surgery

Termino-lateral portacaval anastomosis was performed in rats following the procedure reported by Lee and Fisher [11]. Briefly, rats were put under anesthesia (Ketamine/Xylazine) and a laparotomy was performed to access the abdominal organs. The portal vein was dissected and occluded. The extreme of the portal vein was then connected to a window on the inferior portal vein that was previously obstructed with surgical clips. The PCA was done in less than 20 min. Sham-operated rats were subjected to the same procedure (until the use of the surgical clips) but without cutting any blood vessel. The success of the PCA surgery was ~50% whereas all sham-operated rats survived. Rats with PCA were supplemented with 10% glucose solution the first 2 days after the surgery, and then, they were put in cages with food and water *ad libitum* until the day of their sacrifice (13 weeks later). All operated animals were used in the experimental protocols.

### 2.3. Liver Sampling and Subcellular Fractionation

All rats in each group were decapitated for trunk blood collection. A sample of approximately 3 g was taken from the liver and homogenized in a 10 : 1 proportion in 10 mM Tris-HCl (pH 7.4). Cellular fractionation was done by differential centrifugation as previously reported [12]. Briefly, the homogenate was centrifuged at 1,500 g for 15 min, and the resulting pellet was resuspended and divided into halves for further isolation of plasma membrane fractions. The supernatant was spun at 10,000 g for 15 min to sediment the mitochondrial fraction. The supernatant was ultracentrifuged at 100,000 g for 60 min, resulting in a pellet designated as the microsomal fraction and a supernatant, which was the cytosolic fraction. Both the mitochondrial and microsomal fractions were resuspended in Tris-HCl buffer. All centrifugations were performed at 4°C. The plasma membrane fraction was obtained by centrifuging the first pellet through a Percoll gradient, as described by Loten and Redshaw-Loten [13].

### 2.4. Blood Parameters

Glucose, urea, and triacylglycerides (TAG) were measured by quantitative commercial kits (SPINREACT, Lab-Center, Mexico). Briefly, for glucose determination, glucose oxidase catalyzed the oxidation of glucose to gluconic acid. The formed H_2_O_2_ was detected by the chromogenic oxygen acceptor, phenol 4-aminophenazone, in the presence of peroxidase. Urea in the sample reacted with o-phthaldialdehyde in acid medium forming a colored complex that could be measured by spectrophotometry. Sample TAG was incubated with lipoprotein-lipase, liberating glycerol, and free fatty acids. Glycerol was turned into glycerol 3-phosphate and ADP by glycerol kinase and ATP. Glycerol 3-phosphate was then converted by glycerol 3-phosphate dehydrogenase to dihydroxyacetone phosphate and H_2_O_2_. In the last reaction, H_2_O_2_ reacted with 4-aminophenazone and p-chlorophenol in the presence of peroxidase to give it a red color. Alanine aminotransferase (ALT) and aspartate aminotransferase (AST) were measured as described by Bergmeyer and Bernt [14]. The method used dinitrophenylhydrazine in an acidic medium to develop color that was recorded at 546 nm.

### 2.5. Hematoxylin and Eosin Staining (H&E)

Several segments of the liver (~3 cm^2^) were taken and fixed in formaldehyde 10% buffered at pH 7.4 with potassium phosphate (monobasic and dibasic) for 72 h. The tissue was dehydrated and embedded in paraffin. Paraffin sections were made at 6 *μ*m and stained according to the H&E protocol. Briefly, 1 g of hematoxylin was dissolved in 95% ethyl alcohol, 20 g potassium-aluminum sulfate, and then it was boiled. Hematoxylin is selective for nuclear material. Eosin was dissolved in 80% ethyl alcohol and 0.5 ml acetic acid. Eosin is selective for cytoplasmic material. Slides were sealed with Entellan solution and analyzed in an Olympus microscope CX30. Photographs were evaluated by a pathology expert. To quantify angiogenic activity, 500 liver acini from 5 different sham and PCA samples were inspected to detect the percentage of structures showing ongoing neovascularization.

### 2.6. Electron Microscopy

Liver sections (~1 mm^3^) were cut with sharp razors and fixed in 0.1 mmol/l cacodylate buffer, pH 7.4, containing 2% OsO_4_ and 2.5% glutaraldehyde for 2 h. Dehydration was achieved with ethanol in increasing concentrations: 30%, 50%, 70%, 90%, and 100%. After dehydration, the samples were put into 100% acetone for 30 min and impregnated in a mixture of acetone and Durcupan resin (1 : 1) with gentle rotation overnight. The resin was polymerized by placing the samples in an oven at 600°C for 30 min. The samples were sectioned into 60–80 nm slices in an ultramicrotome and prepared for electron microscopy (JOEL, model 1010). Observations from 6 individual subjects were evaluated for sham and PCA livers. Quantification of the surface cover with mitochondrial corpuscles was done in 12-15 ultrastructural images from sham and PCA samples displaying entire hepatocytes by using ImageJ software (NIH-USA).

### 2.7. Lipid Peroxidation and Conjugated Dienes

Lipid peroxidation (LP) measured *in vitro* was quantified by the 2-thiobarbituric acid method [15] using liver homogenate and subcellular fractions as prooxidative sources. Some modifications to the original method were introduced [16]. Briefly, a sample of the homogenate (~3 mg protein) was incubated for 30 min at 37°C in 1 ml of 0.15 M Tris, pH 7.4; incubation was ended by adding 1.5 ml of 20% acetic acid (adjusted to pH 2.5 with KOH) and 1.5 ml of 0.8% thiobarbituric acid. The samples were kept for 45 min in a boiling water bath, and then 1 ml of 2% KCl was added to each sample. The colored complex was extracted with butanol-pyridine (15 : 1, *v*/*v*) and quantified at 532 nm. Malondialdehyde was used as standard (extinction coefficient: 1.56 Å~105 cm^−1^ M^−1^). LP *in vivo* was determined by conjugated dienes in liver homogenate and subcellular fractions: lipidic fraction was separated with Folch reactive (chloroform-methanol 2 : 1, *v*/*v*); samples were dried, reconstituted in hexane, and measured at 233 nm [17].

### 2.8. Liver Enzymatic Activities

Glutamine synthetase (GS) activity was measured in both liver homogenate and mitochondrial fraction by measuring NADH oxidation in a coupled-enzymatic reaction according to the procedure reported by Kingdon [18]. Briefly, the ATP consumed during glutamine synthesis from glutamate and NH_4_^+^ is regenerated by pyruvate kinase and phosphoenolpyruvate included in the incubations; the resulting pyruvate is reduced to lactate by the added lactate dehydrogenase. This step is coupled to the oxidation of NADH, which is recorded spectrophotometrically at 340 nm. The GS activity was assayed in a final volume of 3 ml using 0.2 mg of total homogenate protein or 0.5 mg of total mitochondrial protein. The reaction was followed for 5 min (to ensure linearity), and the results were expressed as *μ*mol/min/mg using a value of 6.22 as the extinction coefficient for *β*-NADH at 340 nm.

Glutamate dehydrogenase (GDH) activity was measured by an enzyme-coupled reaction according to Schmidt and Schmidt [19]. The redox reaction was followed spectrophotometrically by the oxidation of NADH to NAD^+^ at 340 nm coupled to the conversion of *α*-ketoglutarate to glutamate in the presence of ammonium. Results were calculated using the extinction coefficient of 6220 M^−1^cm^−1^. GDH activity was quantified in liver homogenate as well as in liver mitochondrial and cytosolic fractions.

Caspase-3 is a cysteine-aspartic acid protease with a key role in the executive phase of apoptosis, derived from both extrinsic and intrinsic pathways. The activity of caspase-3 was assayed by a colorimetric method based on the spectrophotometric quantification of chromophore p-nitroaniline (pNA) after cleavage from the labeled substrate DEVD-pNA (Colorimetric kit ABCAM ab39401).

### 2.9. Mitochondrial Fluorescent Probes

MitoTracker (membrane potential), Rhod-2 (matrix calcium), and MitoSOX (superoxide production) were obtained from Molecular Probes, now Thermo Fisher. 50 *μ*g of each dye was diluted in 200 *μ*l of dimethyl sulfoxide. From this stock, 1 *μ*l was taken and diluted in 100 ml of ringer solution. Liver slices (~0.5 cm) were oxygenated and incubated with the dyes for 15 min at room temperature. After incubation, the tissues were fixed in a 4% paraformaldehyde solution for 24 h; subsequently, the samples were placed in different concentrations of sucrose (10, 20, and 30%) for 24 h in each concentration. The tissues were cut to 10 *μ*m in cryostat and observed in the Olympus DP70 fluorescence microscope; then they were quantified at 40X with the ImageJ program.

### 2.10. Indirect Calorimetry

Indirect calorimetry analyses were performed in sham and PCA rats using an OxyletPro System (Panlab Harvard Apparatus, Barcelona, Spain). For this purpose, the animals were placed in individual acrylic cages (Oxylet LE 1305 Physiocage, PANLAB) in a controlled temperature environment (23 ± 2°C) with a photoperiod of 12 h:12 h (lights on at 07:00 h). At week 12 after surgery, a set of 6 rats was acclimated in the Oxylet cages for 48 h before experimental measurements. Oxygen consumption (VO_2_) and carbon dioxide production (VCO_2_) were measured every 12 min for 72 h by an O_2_ and CO_2_ analyzer (Oxylet LE 405-gas analyzer, PANLAB) at a controlled flow rate of 900 ml/min (Oxylet LE 400-air supplier, PANLAB). At each point of analysis, the Metabolism v3.0.01 software (Panlab Harvard Apparatus, Barcelona, Spain) automatically calculated the respiratory quotient (RQ) as the VCO_2_/VO_2_ ratio and energy expenditure (EE) in kcal/day/kg^0.75^as VO_2_ × 1.44 × [3.815 + (1.232 × RQ)], according to the Weir formula [20]. During all procedures, rats had access to food and water *ad libitum*. Activity and food and drink intake were also measured by a continuous recording using Panlab's weight transducer (LE1305 sensor platform, Panlab).

### 2.11. Statistics

Data are presented as mean ± standard error of the mean (SEM). Student's *t*-test was done with the significance threshold set at *P* < 0.05. Statistical analyses were performed with GraphPad Prism 5 and graphs were made with SigmaPlot 10.0.

## 3. Results

### 3.1. Morphometric, Blood, and Hepatic Parameters in PCA Rats

Experimental PCA animals after 13 weeks of surgical procedure showed a variety of morphometric and biochemical alterations, indicating an important shrinkage of the liver and the expected hyperammonemia, but not a clear hepatic necrotic damage (Table 1). PCA promoted a significant reduction in body weight (↓24%) that was even more accentuated in the weight of the liver (↓53%). Therefore, the liver/body weight ratio showed an important reduction (↓37%) in the PCA group. In contrast, no changes were detected in the dry weight of the liver. The most conspicuous alteration measured in the blood was the significant elevation of ammonium (↑90%). No gross signs of hepatic encephalopathy were noted, but fine motor coordination test in a rotating rod was deficient in the PCA rats (data not shown). Table 1 also shows some parameters that indicate the metabolic performance of the liver (circulating glucose and triacylglycerides) as well as markers of cellular liability (serum transaminases and liver caspase-3 activity) and hepatic NH_4_^+^-intracellular handling. The glycemic level was discretely reduced (↓16%) whereas circulating urea and TAG were similar to the values observed in sham rats. Interestingly, PCA rats showed divergent tendencies in ALT and AST blood levels: whereas ALT increased (↑67%), AST decreased (↓32%). The activity of caspase-3 in liver homogenate, as an indicator of ongoing apoptosis, was importantly enhanced (↑47%). Glutamine synthetase (cytosol) and glutamate dehydrogenase (mitochondria), both enzymes involved in hepatic intracellular ammonium-handling, showed significant changes: the 2 enzymatic activities were equally reduced (↓40%).

These data indicate that our experimental surgical model of PCA successfully reproduced the principal alterations reported for this protocol, specifically the enhancement in circulating ammonium levels and an anatomical atrophy of the liver. In addition, the experimental group also showed reduced glycemic values in *ad libitum* conditions suggesting dietary/endocrinal alterations. Modifications in liver glutamine synthetase and glutamate dehydrogenase activities could be related to the ongoing hyperammonemia, and elimination of cellular types by programmed cell death is highly suggestive of metabolic adaptations in the livers from PCA rats. Because only one serum transaminase was elevated (AST), an active necrotic cell disappearance in the liver can be discarded.

### 3.2. Histological and Ultrastructural Characterization in PCA Rats

**Figure 1:**
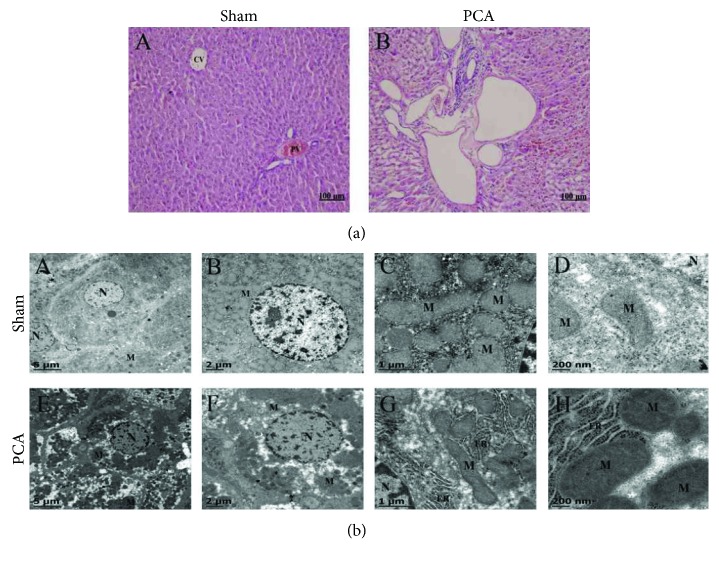
Histological and ultrastructural characterization of hepatocytes. (a) Histological characterization in H&E stained section of the liver. (A) Liver from sham operated rat. CV: central vein; PV: portal vein. (B) Experimental animal with PCA showing formation of new blood vessels. (b) Ultrastructural characteristics of hepatic cells. (A–D) Normal hepatocyte of sham-operated rats. (F–H) Hepatocyte cells of PCA-operated rats. N: nucleus; M: mitochondrial; ER: endoplasmic reticulum. Images are representative of 6 independent experimental observations.

It was evident in the histological study using H&E staining that PCA livers showed a remarkable new feature: all operated animals showed an extensive process of neovascularization (approximately 1 in every 20 liver acini) that made it difficult to distinguish the usual hepatic zonation of pericentral and periportal regions (Figure 1(a), B). Hepatocytes with morphological characteristics of apoptotic activity were also detected, supporting the finding of elevated caspase 3 activity in the liver homogenate (Table 1). Images of electron microscopy displayed some ultrastructural abnormalities: (1) the nuclei did not look as turgid as the nuclei in hepatocytes from sham rats; (2) hepatocytes from PCA rats showed less mitochondrial material (~30%) evidencing extensive zones of protoplasm filled with small vacuoles; and (3) widespread endoplasmic reticulum cisterna with numerous ribosomes (Figure 1(b), E–H).

Analysis of the cellular morphology of PCA rat livers highly suggests that the hepatic gland is adapting to a new equilibrium in response to the hemodynamic challenge that the portal bypass involves: an active angiogenic process that is coincident with an altered subcellular structure, fewer mitochondria, and ongoing apoptotic activity. However, no evidence of necrotic cell destruction was detected.

### 3.3. Liver Prooxidant Reactions in PCA Rats

**Figure 2:**
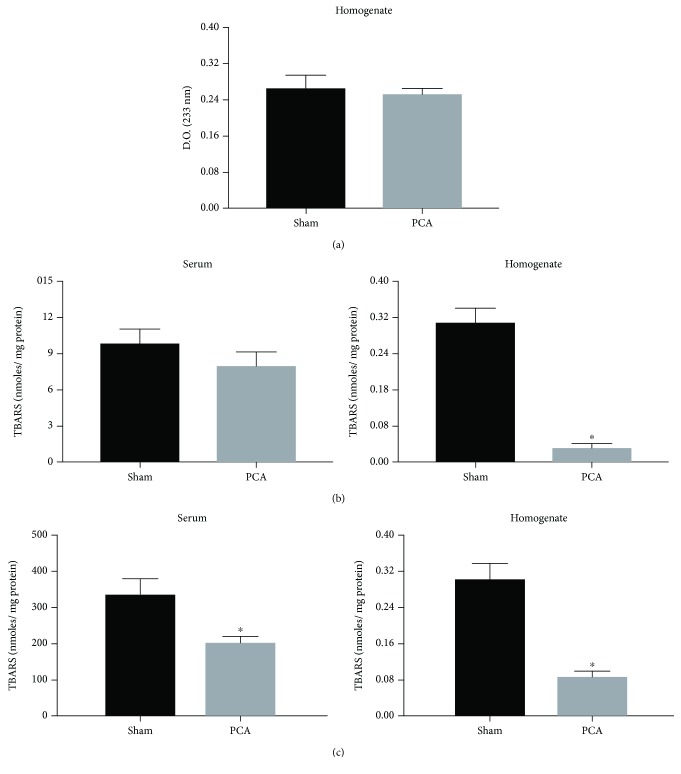
Prooxidant reactions in the serum and liver homogenate. (a) Conjugated dienes (not measured in serum). (b) Basal TBARs. (c) TBARs supplemented with FeSO_4_ 50 *μ*M. Data obtained from sham and PCA rats after 13 weeks of surgical procedures. Values represent mean ± SEM of 12-15 independent experimental observations; *P* < 0.05.

**Figure 3:**
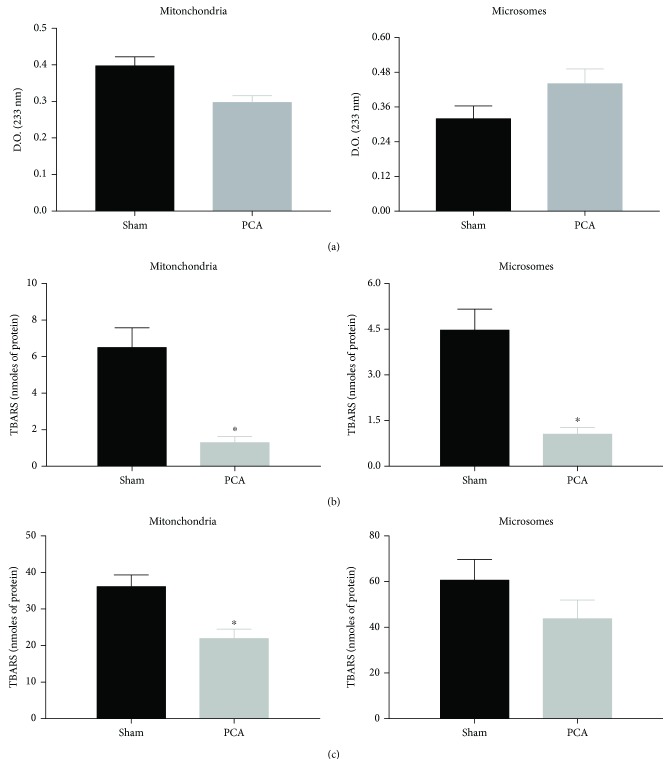
Prooxidant reactions in mitochondrial and microsomal fractions of the liver. (a) Conjugated dienes. (b) Basal TBARs. (c) TBARs supplemented with FeSO_4_ 50 *μ*M. Subcellular fractions were obtained by differential centrifugation (see Methods). Data obtained from sham and PCA rats after 13 weeks of surgical procedures. Values represent mean ± SEM of 12-15 independent experimental observations; *P* < 0.05.

**Figure 4:**
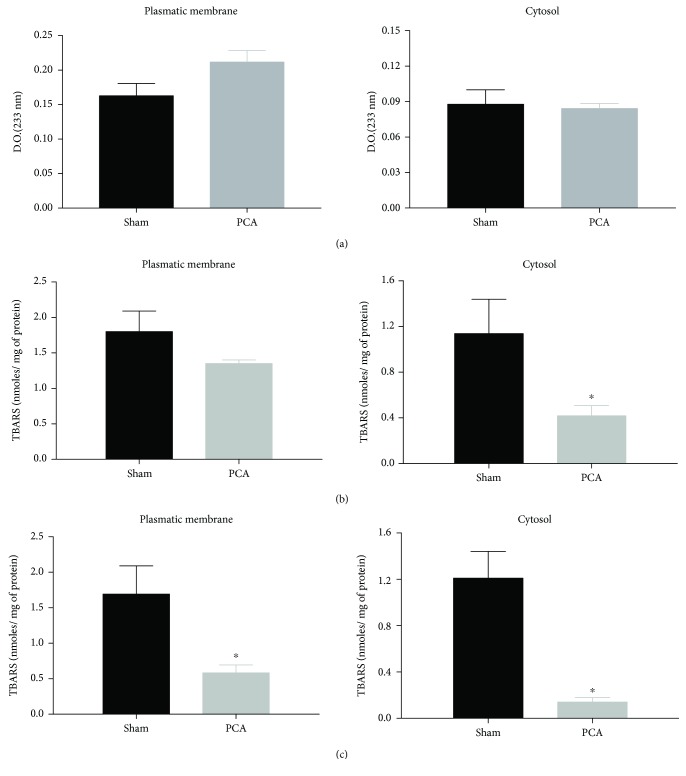
Prooxidant reactions in the plasma membrane and cytosolic microsomal fractions of the liver. (a) Conjugated dienes. (b) Basal TBARs. c) TBARs supplemented with FeSO_4_ 50 *μ*M. Subcellular fractions were obtained by differential centrifugation (see Methods). Data obtained from sham and PCA rats after 13 weeks of surgical procedures. Values represent mean ± SEM of 12-15 independent experimental observations; *P* < 0.05.

Despite the suggestive morphological features of cellular stress observed by electron microscopy (Figure 1(b), E–H), the protocol of experimental PCA for 13 weeks was not associated with any increase in 2 different markers of prooxidant reactions in the livers of PCA rats, namely, conjugated dienes and TBARs (basal levels and Fe^2+^-supplemented) (Figures 2–4). Indeed, not a single increment in oxidative markers in PCA rats was observed in the whole liver homogenate and all subcellular fractions tested.  Figure 2 shows that conjugated dienes in liver homogenate from PCA rats were similar to the values recorded in sham rats. No conjugated dienes were determined in the serum. In contrast, basal levels of TBARs in liver homogenate from PCA rats showed a very notorious reduction (↓87%) that was also present when the assay was supplemented with Fe^2+^ (↓71%). Basal TBARs did not show any change in the serum of PCA rats, but they depicted a significant reduction in the presence of Fe^2+^ (↓42%).  Figure 3 displays the prooxidant status in the mitochondrial and microsomal fractions of the liver. In both fractions, the changes in conjugated dienes were discreet and did not reach statistical significance. However, in basal TBARs, values of mitochondrial and microsomal fractions from PCA rats showed evident diminutions: ↓82% in the mitochondria and ↓70% in microsomes. With the Fe^2+^ supplementation, the TBAR value decreased in the mitochondrial fraction (↓43%), whereas in the microsomal fraction it showed only a tendency to be reduced but without statistical significance. Data from the liver plasma membrane and cytosolic fractions from PCA rats are shown in Figure 4. As with the other subcellular fractions, conjugated diene levels were very similar between sham and PCA rats in these fractions. Similar to the data from Figures 2 and 3, basal TBARs showed a reduction: ↓22% in plasma membrane and ↓59% in cytosol. A more notorious decrease was observed when Fe^2+^ was present in the assay. In this condition, the reduction in the plasma membrane fraction was ↓67%, whereas in the cytosolic fraction it was ↓87%.

Hence, despite the histological and cellular alterations in the liver of PCA rats, the structural abnormalities were not coincident with an elevation in the levels and activity of prooxidant markers.

### 3.4. Mitochondrial Fluorescent Probes

**Figure 5:**
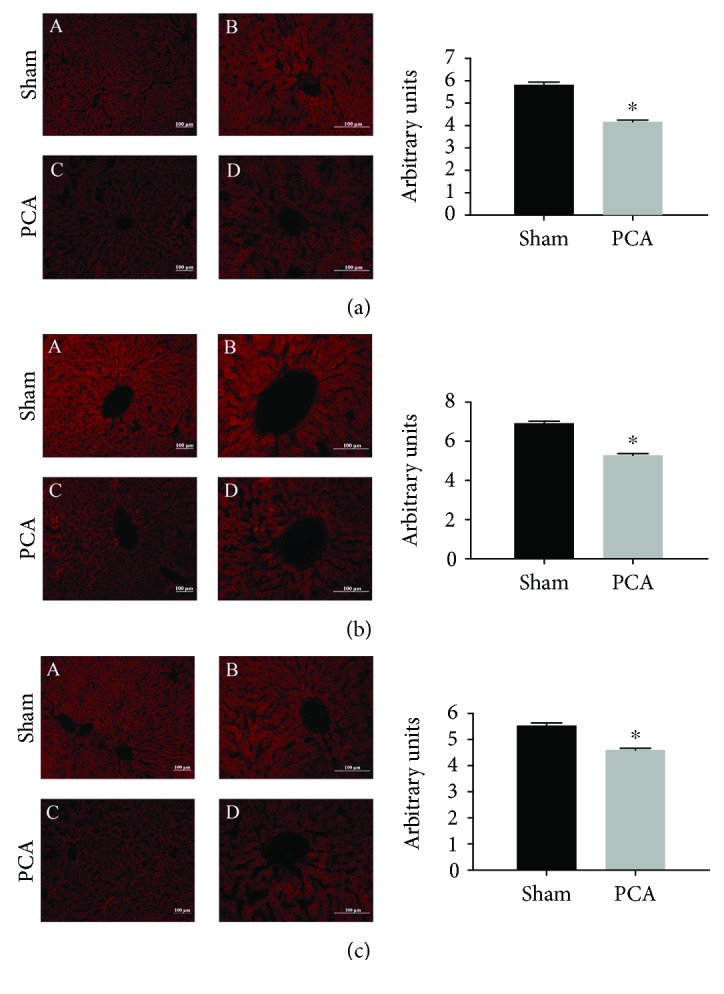
Mitochondrial parameters by fluorescent dyes. (a) MitoTracker for assessment of mitochondrial membrane potential ΔΨ_*m*_. (b) Rhod-2 to measure mitochondrial calcium. (c) MitoSOX for mitochondrial reactive oxygen species (ROS) detection. Histograms at the right side show the quantification of the fluorescent signals, expressed as average ± SEM. Statistical analysis was done by Student's *t*-test with *P* < 0.05; ^∗^ means a significant difference. Graphics are representative of 12-15 independent experimental observations.

Experiments with MitoTracker, Rhod-2, and MitoSOX were done to study the functional and prooxidant status of the hepatic mitochondria in PCA rats. Data are shown in Figure 5. In the 3 panels the results were comparable, a significant diminution of 21-25% in the fluorescent signal.

Taken together, the ultrastructural analysis of the mitochondrial presence and the functional mitochondrial probes resulted in a similar degree of reduction suggesting that PCA surgery affects more the liver mitochondrial number than their functional status.

### 3.5. Metabolic Performance in PCA Rats

**Figure 6:**
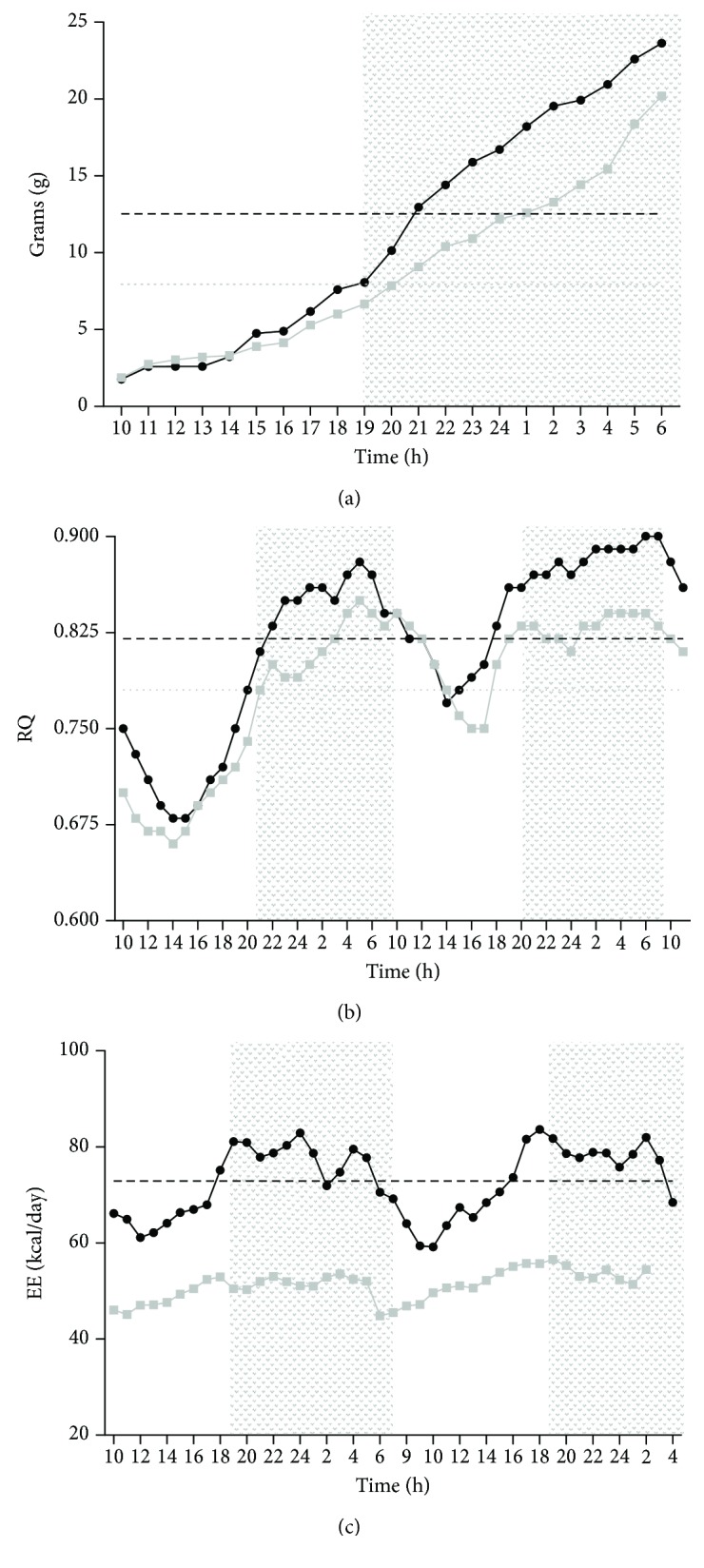
Food intake, respiratory quotient, and energy expenditure in rats with PCA. Data were collected over a period of 1-2 days using metabolic cages (PANLAB, Spain). Darker section depicts period with light off. (a) Cumulative food intake, expressed in g, during 24 h. (b) Respiratory quotient (ratio of CO_2_ produced by the O_2_ consumed) recorded for 2 days. (c) Energy expenditure recorded for 2 days. Data from panels (b) and (c) were recorded every 12 min and the results shown in the graphics correspond to values calculated with the average of every hour. To make simpler the display of the data, only the mean of 6 independent experimental observations are represented; SEM values (corresponding from 8 to 13% of the mean) were omitted. PCA rats showed significant reduction in food intake and respiratory quotient during the dark period and along the 24 h recording in energy expenditure.

By using open-flow indirect calorimetry, we evaluated in PCA rats the individual components of energy demand such as the respiratory coefficient, cumulative food intake, water drinking, and ambulatory activity. The results involving 24-48 h of continuous recording are depicted in Figure 6. It can be seen that rats after 13 weeks of PCA surgery displayed a reduced pattern of metabolic performance, evidenced by lower energy expenditure and lower respiratory quotient in comparison to sham rats (Figures 6(a) and 6(b)). In both, the diminution was more evident during the dark period which is the time nocturnal rodents are awake. The reduced metabolic activity shown by the PCA rats was accompanied by a significant decrease in the 24 h pattern of food intake (Figure 3(c)). Again, the difference was more accentuated during the dark period. No changes related to the experimental surgery were observed in the daily pattern of water drinking and ambulatory activity (data not shown).

## 4. Discussion

### 4.1. Experimental Protocol

PCA as an experimental model has been mainly focused on characterizing brain alterations associated with hyperammonemic encephalopathy [3]. In contrast, much fewer reports exist regarding the modifications that take place in the liver after the surgery. In particular, the portal bypass involved in PCA importantly influences the uptake, processing, and distribution of nutrients that take place in the hepatic gland. Therefore, it is expected that the liver oxidant metabolism becomes affected as part of the bioenergetic adaptations that are triggered by the loss of the normal hepatic hemodynamics. In this context, previous reports have shown that oxidative stress is developed in cerebral tissues because of the PCA surgery related with high levels of ammonium [21], but none exists regarding the prooxidant status in the liver. Other striking feature associated to the PCA was the notorious reduction of the liver size (Table 1), reduction that was accompanied by an increased apoptotic activity, but at the same time by an enhanced formation of blood vessels without active necrosis (Table 1 and Figure 1(a)). Previous reports have associated the altered hepatic nutrient arrival caused by the portal deprivation to an active angiogenesis [22]. Damage to the liver cells was discreet at most, since only serum ALT was elevated whereas circulating AST was reduced, and urea levels did not change. Active necrotic cell death is usually associated to the elevation of both transaminases in the serum [23]. All these data are suggestive of an emergent balance in which the morphological changes observed in the liver coexist with a functional hepatic activity.

An important parameter to take into consideration in the PCA model is the elapsed time postsurgery. Because the protocol involves a progressive adaptation and modification of the metabolic and physiological activities, it is necessary to clearly establish the time in which the experiments are done. For example, 3.5 months after PCA surgery were necessary to promote discreet changes in the properties of the liver mitochondria [24], whereas only 3 weeks were enough to affect gene expression and lipogenic activity in the adipose tissue [25]. In particular, our experiments were done after 13 weeks of PCA surgery to ensure a well-established chronic adaptation in the metabolic and oxidative capacities of the liver in response to the new hemodynamic circumstances.

### 4.2. Histological and Cellular Changes

The principal consequence of PCA is to disconnect the portal circulation to the liver. Indeed, this is a major manoeuvre to drastically change the hepatic hemodynamics. One of the most notorious outcomes was the proliferation of new blood vessels in the liver parenchyma (1 in every 20 liver acini in PCA rats). Proangiogenic factors in the liver are numerous and very complex in their mechanisms of action. Some proangiogenic factors are angiopoietin, VEGF, FGF, EGF, and thrombospondin; the role played by the stellate cells is also relevant [26]. Further experiments are needed to learn which factors are involved in the proangiogenic response observed in the PCA livers after 13 weeks of surgery (Figure 1(a)).

Another conspicuous modification detected in the ultrastructural study of the hepatocytes from PCA livers was the reduced presence of mitochondrial bodies (↓30%) (Figure 1(b)). A possible explanation to this effect is that the disturbed oxygenated blood flow resulting from the PCA (portal blood is more oxygenated than the blood circulating in the central vein) could affect the hepatic oxidative metabolism and thus the hepatic zonation. The presence of fewer mitochondria could explain the generalized and similar reduction observed with the 3 mitochondrial fluorescent probes (MitoTracker, MitoSOX, and Rhod-2) tested in Figure 5. The coincidence in the degree of diminution of mitochondrial corpuscles and the functional fluorescent probes strongly suggest that PCA alters mainly the mitochondrial number but not their properties. The interesting possibility that hepatic zonation (metabolically specialized hepatocytes in the periportal and pericentral regions) could be modified by the PCA remains to be tested.

### 4.3. Prooxidant Reactions

In our experiments, we measured 2 markers of oxidative stress in liver subcellular fractions and serum (Figure 2): (1) conjugated dienes as a measure of an ongoing lipid peroxidation, also considered by some authors as an “in vivo” lipid peroxidation and (2) TBARs, in basal conditions and Fe^2+^-supplemented, to evaluate the prooxidant potential of the sample. In addition, the tests were done in liver homogenate and subcellular fractions, since it has been demonstrated that lipid peroxidation can be increased differentially only in some endomembranes, according to the physiological or pharmacological condition studied [12].

Concerning the oxidative activity, PCA surgery involved a clear reduction in the prooxidant activity, confirmed by 2 different markers of lipid peroxidation (Figure 2). These data ruled out a condition of oxidative stress in the liver and serum of the rats after 13 weeks of PCA surgery, despite the evident histological and ultrastructural alterations such as the process of neovascularization, the abnormalities in the nuclear morphology, the reduced mitochondrial number, and the ongoing apoptosis (Figure 2). A putative interpretation to the lower lipoperoxidative activity in the subcellular fractions of the liver from PCA rats is to consider that the liver could be adapting a hypofunctional state because of the reduced metabolic demands caused by the portal deprivation [22].

These results could also be explained supposing that the appearance of new blood vessels is a regulated process. Hence, the prooxidant reactions are reduced as an adaptation for the liver to properly function despite an ongoing apoptosis. In particular, the reduction of TBAR values in the livers from PCA rats in the presence of Fe^2+^ is suggestive of minor availability of unsaturated acyl groups in the membrane phospholipids. This situation could be related to a hepatocyte membrane restructuration, in both plasma membrane and endomembranes.

Further experiments are needed to clarify the molecular mechanisms underlying these possibilities.

### 4.4. Metabolic Performance

PCA is an experimental protocol that greatly affects the wellness of rats under the condition of our study and after 13 weeks of the surgical procedure. Data in Table 1 showed a significant reduction in body weight in PCA rats. This alteration was sustained by the confirmation of a reduced daily food intake (Figure 6(a), especially during the dark period). Interestingly, this diminution was not associated with alterations in the ambulatory behavior of PCA rats (data not shown). PCA rats showed an altered hepatic histology since it is coincident with an ongoing angiogenic and apoptotic activities (Table 1 and Figure 1(a)). This circumstance associated with the hyperammonemic condition and the reduced food intake may be related to the minor energy expenditure and respiratory quotient (Figures 6(b) and 6(c)) as well as to the lower glycemia (Table 1) recorded in PCA rats. The altered metabolic performance shown by the PCA rats was not associated with their lighter weight since the energy expenditure and the respiratory quotient were similar in intact rats weighing ~430 g and ~320 g (data not shown). Taken together, the parameters obtained in the metabolic cages are indicative of a hypometabolic status in the PCA rats that provides novel perspectives regarding this experimental surgical model.

## 5. Conclusions

After 13 weeks of surgery, experimental PCA promoted hyperammonemia and a reduction of liver mass. A process neovascularization and apoptosis in the liver were evident whereas hepatocytes showed diminished mitochondrial content. However, no active necrotic cell destruction was detected. Lower levels of blood glucose were detected. Hepatic prooxidant reactions measured as TBARs showed a notorious reduction in the PCA group, especially in whole homogenate as well as in microsomal and mitochondrial fractions (at basal conditions), and in whole homogenate and cytosolic fraction (supplemented with Fe^2+^). Changes in conjugated dienes were discreet. Active energy expenditure as well as food intake was reduced in the PCA group. Overall, data are suggestive of a hypometabolic response that is associated with a lower prooxidant reaction in the liver of PCA rats after 13 weeks of surgery.

## Figures and Tables

**Table 1:** Morphometric and biochemical parameters in rats with PCA.

	Sham		PCA	
	Mean	SEM	Mean	SEM
Body weight (g)	444.0	15.0	335.6^∗^	16.0
Liver weight (g)	15.5	0.7	7.3^∗^	0.4
Liver dry weight (%)	71.7	0.8	72.4	1.1
*Blood*				
Ammonium (*μ*g/ml)	2.1	0.2	4.0^∗^	0.4
Urea (mg/dl)	56.8	2.9	51.2	1.6
Glucose (mg/dl)	135.4	7.0	113.3^∗^	3.2
TAG (mg/dl)	46.4	6.6	44.5	4.2
ALT (U/l)	10.6	1.1	17.7^∗^	3.0
AST (U/l)	282.5	13.6	191.8^∗^	2.9
*Liver*				
Glutamine synthetase (cytosol) (*μ*moles NADH/min/mg)	10.9	1.4	6.6^∗^	1.2
Glutamate dehydrogenase (mitochondria) (*μ*moles NADH/min/mg)	317.6	40.6	190.5^∗^	12.6
Caspase 3 (absorbance at 405 nm)	1.5	0.3	2.2^∗^	0.2

Body and liver weight, blood parameters, and liver enzymatic activities were determined in rats with PCA 13 weeks after surgery. Blood determinations were performed in the serum and liver determinations were done in homogenate and subcellular fractions. TAG: triacylglycerols; ALT: alanine aminotransferase; ASP: aspartate aminotransferase. Significant differences were detected in cytoplasmic glutamine synthetase activity and in mitochondrial glutamate dehydrogenase activity. No changes were detected in the activity of both enzymes in the liver homogenate. Values correspond to 7-12 sham rats and 15-19 PCA rats. Statistical significance (^∗^) was calculated by the Student *t*-test; *P* value was set at 0.05.

## Data Availability

All the experimental protocols, material, and data obtained to support the findings and to sustain the conclusions of this study are available from the corresponding author upon request.
